# Catheter Ablation Improves Ventilatory Efficiency in Atrial Fibrillation‐Mediated Cardiomyopathy

**DOI:** 10.1111/jce.16606

**Published:** 2025-02-21

**Authors:** Nikhil Ahluwalia, David Bruce, Ashley Ashdown, Fabrizio Focacci, Hakam Abbass, Shohreh Honarbakhsh, Anthony Chow, Mehul Dhinoja, Ross J. Hunter, Steffen Petersen, Guy Lloyd, Richard J. Schilling

**Affiliations:** ^1^ St Bartholomew's Hospital, Barts Heart Centre London UK; ^2^ Queen Mary University of London London UK

**Keywords:** atrial fibrillation, catheter ablation, exercise oscillatory ventilation

## Abstract

**Background:**

Exercise Oscillatory Ventilation (EOV) and a steep ventilatory efficiency (VE/VCO2) slope are features of ventilatory inefficiency on cardiopulmonary exercise testing (CPET), both associated with poor prognosis in patients with heart failure (HF). The prevalence in patients with co‐existent atrial fibrillation (AF) and the impact of catheter ablation (CA) is unknown.

**Objectives:**

To characterize ventilatory inefficiency in patients with persistent AF and Left Ventricular Systolic Dysfunction (LVSD) and assess the impact of CA.

**Methods:**

Patients with persistent AF and Left Ventricular Ejection Fraction (LVEF) < 50% undergoing first‐time CA were prospectively enrolled. Echocardiography and CPET were performed at baseline and 6 months post‐CA. EOV was defined using the Kremser–Corrà criteria, and VE/VCO2 slope gradient > 30 was considered abnormal.

**Results:**

A total of 53 participants were enrolled (mean LVEF of 34 ± 9%). A total of 10 (19.2%) exhibited EOV at baseline. These patients had larger indexed left atrial (41.6 ± 13.1 mL/m^2^ vs. 33.3 ± 9.3 mL/m^2^, *p* = 0.03) and ventricular volumes [65.7 mL/m^2^ (57.1, 89.0) vs. 46.7 mL/m^2^ (39.8, 61.4), *p* = 0.03]. The partial pressure of end‐tidal carbon dioxide (P_ET_CO_2_) at peak exercise increased (33.7 ± 6.1 mmHg to 41.2 ± 5.8 mmHg, *p* < 0.001) and correlated with improvement in HF symptoms (*p* = −0.003) and objective HF markers. A total of 25 (48.1%) had an abnormal VE/VCO2 gradient. The EOV pattern resolved in eight (80%) participants due to a reduction in EOV burden (71.1 ± 11.9% vs. 48.8 ± 14.8%, *p* = 0.006) and the component amplitude of minute ventilation cycles (2.6 L/min (2.5,3.2) vs 2.2 L/min (1.8,2.6), *p* = 0.028). Fewer patients had an abnormal VE/VCO2 gradient after CA [25 (48.1%) vs. 16 (34.0%), *p* = 0.004].

**Conclusions:**

Ventilatory inefficiency is common in patients with AF and LVSD. CA improves both EOV and VE/VCO2 in AF‐induced cardiomyopathy. Improvement in P_ET_CO_2_ is also seen and correlates with HF symptom burden.

**Abbreviations**
AF: atrial fibrillation
AIC: AF‐induced cardiomyopathy
AT: atrial tachycardia
AUROC: area under the receiver operating characteristic
BMI: body mass index
CA: catheter ablation
COPD: chronic obstructive pulmonary disease
CPET: cardio‐pulmonary exercise test
EOV: exercise oscillatory ventilation
HF: heart failure
LAVi: left atrial indexed volume
LV: left ventricle
LVEF: left ventricular ejection fraction
LVSD: left ventricular systolic dysfunction
MLWHQ: minnesota living with heart failure questionnaire
NT‐proBNP: N‐terminal pro‐B‐type NATRIURETIC PEPTIDe
PETCO2: partial pressure of end‐tidal carbon dioxide
RER: respiratory exchange ratio
VE: minute ventilation
VE/VCO2: minute ventilation‐carbon dioxide production relationship

## 1 Introduction

Inefficient ventilation can manifest in heart failure (HF) as ventilation‐perfusion mismatching or a periodic breathing pattern during exercise. These phenomena can be identified using cardio‐pulmonary exercise testing (CPET) as a steep minute ventilation‐carbon dioxide production (VE/VCO2) slope gradient and exercise oscillatory ventilation (EOV). EOV is a periodic breathing pattern with characteristic cyclic fluctuation in minute ventilation and tidal volume during the exercise phase. An elevated VE/VCO2 may reflect reduced systemic perfusion in HF, resulting in an increased ventilation with respect to CO2 clearance, EOV results from a dysregulation of the feedback control of ventilation, and the mechanisms are only partially understood. Both parameters are associated with poor prognosis in HF and EOV is the strongest predictor of survival on CPET, outperforming peak VO2 but there are fewer data describing this [1].

Atrial fibrillation (AF) complicates 20%–45% of patients with HF and is independently associated with an increased risk of HF hospitalization and mortality [2]. Despite its prognostic value, the phenomenon and its prevalence in patients with co‐existent AF have not been studied.

EOV is a marker of advanced HF, although some HF treatments have demonstrated a reversibility of the phenomenon [3, 4, 5]. Catheter ablation (CA) of AF is associated with a greater improvement in clinical outcomes in patients with end‐stage HF than medical therapy alone and can ameliorate the clinical course. The effect on EOV and the underlying mechanisms are unknown. This study aims to report the prevalence of ventilatory inefficiency in patients with AF and HF and evaluate the impact of CA on the EOV pattern and patient‐reported dyspnoea symptoms.

## 2 Methods

### 2.1 Study Design

Patients with persistent AF and LVSD (LVEF < 50%) undergoing first‐time CA at the study institution were invited to enroll in this prospective, observational study. The study received approval from the UK National Research Ethics Committee (21/SW/0135) and was registered on clinicaltrials.gov (NCT04987723). Persistent AF was defined as more than 7 days of continuous, sustained AF. Patients with a recent reversible cause of AF, any change in treatment for LVSD, or a left atrial indexed volume (LAVi) > 50 mL/m^2^ were excluded. Trans‐thoracic echocardiography, serum NT‐proBNP and cardiopulmonary exercise testing (CPET) were performed at baseline and 6 months after the final CA.

### 2.2 Cardio‐Pulmonary Exercise Test

CPET was performed on a semi‐recumbent cycle ergometer (ERG 911 S/L, Schiller, Switzerland) to evaluate metabolic capacity using a compatible cart (Quark, Cosmed, Italy). Minute ventilation (VE), oxygen saturation, and carbon dioxide production were monitored. Following gas and syringe volume calibration, resting parameters were recorded whilst stationary for up to 3 min. A further 3‐min warm‐up followed, and then an incremental exercise period wherein the work rate was increased by 10–20 W per minute till exhaustion, aiming for 8–10 min of exercise and a respiratory exchange ratio of > 1.0.

**Figure 1:**
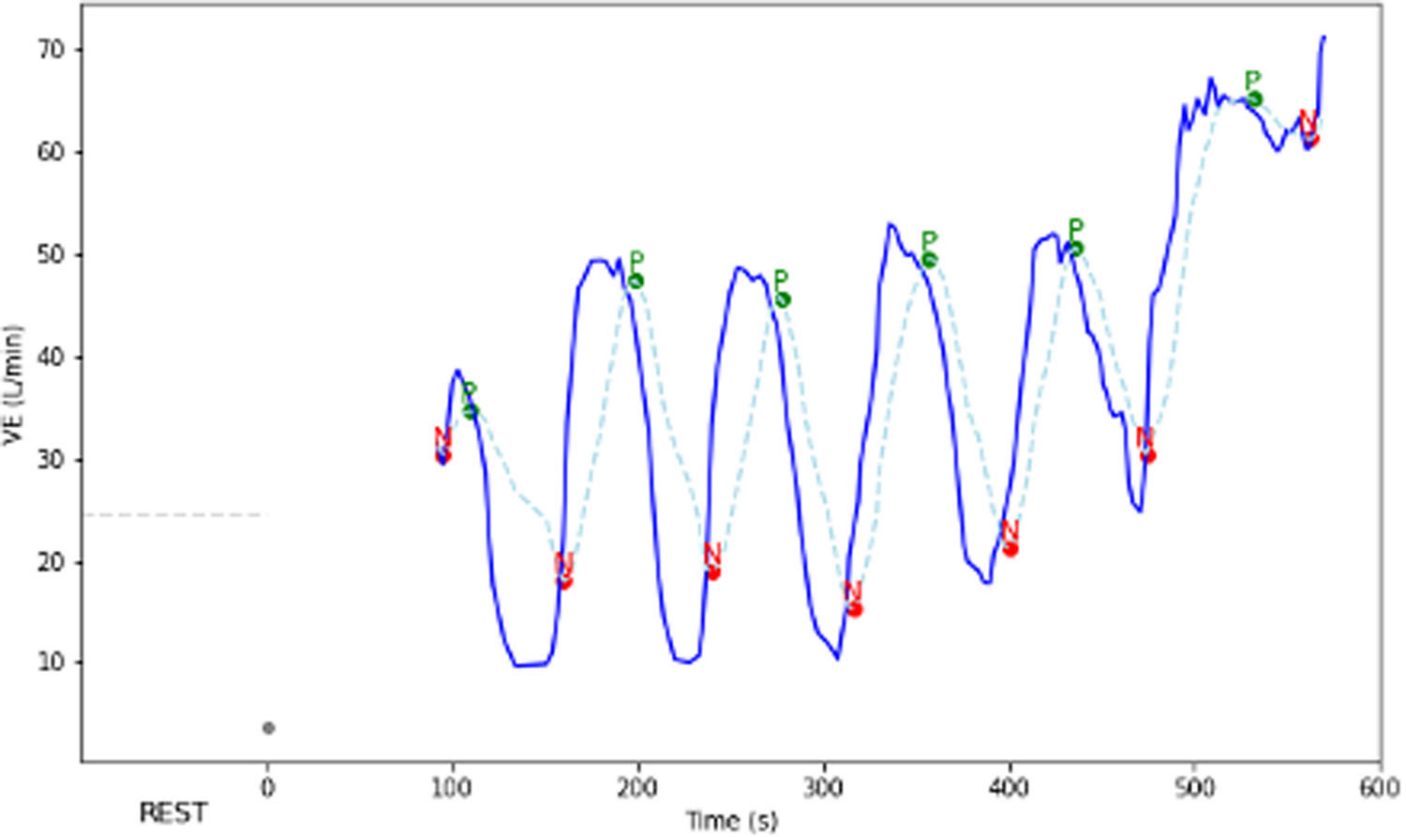
Dynamic change in minute ventilation (VE) throughout the exercise phase of the cardiopulmonary exercise test in an example patient with exercise oscillatory ventilation. The raw VE value (dark blue) and rolling 10‐s average Ve value (dashed light blue) are plotted. The local peaks (P) and nadirs (N) are annotated on the smoothed plot. The amplitude (a) is the VE difference between the cycle peak and the average of the adjacent nadirs. The mean VE during the rest phase (dashed gray line) is used to calculate the threshold deviation (15% of mean VE, represented as the black arrowheaded line at Time = 0) for the EOV calculations. EOV calculation: The cycle length (CL) is the time between consecutive nadirs. The EOV burden is calculated as the sum of the CLs of cycles with an amplitude greater than the threshold deviation (15% of the mean VE during the rest phase). The EOV burden is expressed as a percentage of the total duration of the exercise phase. EOV is diagnosed when the EOV burden is greater than 0.60.

The Kremser–Corrà definition of EOV as outlined in the International Consensus document was used; cyclical fluctuation in the VE graph of greater than 15% of the mean VE during the resting period for greater than 60% of the exercise test [6]. The 10‐s rolling averaged VE was plotted to identify fluctuations. The average of consecutive nadirs was used to calculate cycle amplitude (Figure 1). The composite quantitative features were the EOV burden (the duration of cycles meeting EOV criteria as a percentage of total exercise time), average cycle amplitude and average cycle length (CL) were also measured. A bespoke script (Python 3.10.9) was developed to handle the raw data files outputted by the cart and determine the EOV status and component parameters. This is available on request.

The VE/VCO2 slope gradient was calculated using linear regression of the relationship from start to peak of the exercise phase [6]. A gradient steeper than 30 was in keeping with ventilatory inefficiency [6]. Peak VO2, averaged over the final 30 s of exercise, was also measured. The partial pressure of end‐tidal carbon dioxide (P_ET_CO_2_) was measured.

The MLWHQ was completed at baseline and follow‐up to characterize symptom burden. The 21‐question survey provides a total score (range: 0–105, from best to worst Health‐Related Quality of Life).

### 2.3 Catheter Ablation

The CA strategy was at the discretion of the clinical operator, with wide‐area circumferential radio‐frequency ablation of the pulmonary veins with left atrial electro‐anatomical mapping [Ensite X, Abbott Inc, USA] as a minimum. Antiarrhythmic medication was routinely discontinued at 3 months. Oral anticoagulation and guideline‐directed HF medications were routinely continued beyond primary endpoint evaluation. AF recurrence was defined as AF detection. Re‐ablation for symptomatic drug‐refractory recurrences of AF and atrial tachycardia was at the discretion of the referring clinician after at least 3 months from the index procedure. Re‐do CA re‐started follow‐up assessment time points within this study.

### 2.4 Statistics

The Shapiro–Wilk test was used to determine whether data was normally distributed. Continuous variables were analysed using a two‐tailed independent *t*‐test for normally distributed data or the Mann–Whitney *U*‐test for non‐normally distributed data. The Chi‐squared test was used for unpaired categorical variables and McNemar's test if paired. Normally distributed data was presented as mean ± standard deviation, and non‐normally distributed data as median (interquartile range). The Pearson Correlation coefficient was calculated for normally distributed continuous variables and the Spearman Correlation coefficient for non‐linear relationships and non‐normal variables. Area Under the Receiver Operating Characteristic (AUROC) curve analysis was performed to evaluate the predictive value of continuous variables in relation to dichotomous outcomes. Linear regression was applied for continuous outcome measures. Cases with missing data were excluded from the respective analysis. A *p*‐value < 0.05 was used to determine significance.

## 3 Results

### 3.1 Study Population

One hundred and one patients with LVEF < 50% were screened, and 53 were enrolled. The mean age was 59.1 ± 11.5 years, and eight (15.1%) participants were female. Their BMI was 30.0 ± 5.3 kg/m [2]. The mean LVEF was 34 ± 9%, with 22 (41.5%) patients having severe LVSD (≤ 35%) during AF. The mean MLWHQ score was 43 ± 25 (/105). Two (37.7%) participants had a diagnosis chronic obstructive pulmonary disease (COPD) and no other participant had known respiratory pathology. A total of 51 (96.2%) participants reported physical symptoms at baseline, with 48 (90.6%) reporting shortness of breath. A total of 52 (98.1%) participants were on beta‐blockers, and 51 (96.2%) were on renin‐angiotensin system inhibitors. Median ambulatory NT‐proBNP at baseline was 917 pg/mL (554, 1663).

### 3.2 Metabolic Parameters During AF

All participants achieved an RER ≥ 1.0, and the test was stopped in all cases due to patient‐reported exhaustion. The mean exercise time on CPET was 8.7 ± 2.4 min, with a maximum power of 139 ± 52 Watts. Peak VO2 was 1714 ± 574 mL/min with an indexed Peak VO2 of 18.6 ± 7.2 mL/kg/min^−1^. The median VE/VCO2 slope was 32.4 ± 9.2 mL/kg/min and 25 (48.1%) participants had inefficient ventilation, evidenced by an abnormal VE/VCO2 slope gradient.

The median EOV burden was 41% (32, 53) with a median cycle amplitude of 1.9 L/min (1.3, 2.4). The EOV burden and amplitude correlated with LVEF on echocardiography (*r* = −0.30, *p* = 0.03 and *r* = −0.38, *p* = 0.006, respectively). The P_ET_CO_2_ at peak exercise was 33.7 ± 6.1 mmHg. This parameter correlated with HF symptom burden by MLWHQ score (*r* = −0.41, *p* = 0.003), whereas established markers of HF severity such as LVEF, NTpro‐BNP level and peak VO2 did not. The P_ET_CO_2_ at rest was 30.4 ± 3.5 mmHg and associated with the LVEF (*r* = 0.34, *p* = 0.015).

A total of 10 (19.2%) participants demonstrated an EOV pattern during baseline CPET with a VE/VCO2 slope, and 35.8 ± 12.6 mL/kg/min had a peak VO2 of 63.8 ± 13.3% of their predicted VO2 max. They had significantly larger indexed left atrial volumes (41.6 ± 13.1 mL/m^2^ vs. 33.3 ± 9.3 mL/m^2^, *p* = 0.03) and left ventricular volume during both diastole [65.7 mL/m^2^ (57.1, 89.0) vs. 47.1 mL/m^2^ (40.1, 61.0), *p* = 0.03] and systole [43.7 mL/m^2^ (33.7, 66.5) vs. 30.4 mL/m^2^ (24.2, 38.6), *p* = 0.03] (Table 1). Patients with EOV had lower peak E‐wave velocity (52 ± 12 cm/s vs. 73 ± 21 cm/s, *p* = 0.006), but no difference in mitral deceleration time was seen (*p* = 0.85). There was no significant difference in cardiac function parameters or HF medications.

**Table 1:** Distribution of demographic parameters in participants with Exercise Oscillatory Ventilation (EOV) pattern (EOV‐positive) at baseline compared to participants without (EOV‐negative).

	EOV‐positive	EOV‐negative	*p*‐value
Age, years	56.6 ± 14.7	60.0 ± 10.8	0.427
Male, *n* (%)	10 (100)	34 (81.0)	0.311
Hypertension, *n* (%)	3 (30)	19 (45.2)	0.603
Ischaemic heart disease, *n* (%)	1 (10)	7 (16.7)	0.970
Diabetes, *n* (%)	2 (20)	6 (14.3)	1.000
BMI, kg/m^2^	30.3 ± 4.5	30.0 ± 5.6	0.837
LVEF, %	31 ± 10	35 ± 9	0.137
LAVI, mL/m^2^	41.6 ± 13.1	33.3 ± 9.3	0.027
Mean HR, bpm	88.1 ± 17.8	83.8 ± 14.2	0.447
NT‐proBNP, pg/mL	823.0 (556.5, 1381.75)	918.0 (575.0, 1775.0)	0.915
AF duration, months	10 (7, 14)	10 (5, 16)	0.808
CHADS2VASc score	1.5 (1.0, 2.8)	2.0 (1.0, 3.8)	0.231
LVEDVi, mL/m^2^	65.7 (57.1, 89.0)	47.1 (40.1, 61.0)	0.031
LVESVi, mL/m^2^	43.7 (33.7, 66.5)	30.4 (24.2, 38.6)	0.034
Max HR, bpm	148.0 ± 16.24	137.35 ± 18.6	0.102
MLWHQ score (/105)	42 ± 23	42 ± 25	0.977
Test duration (mins)	8.4 ± 2.4	8.7 ± 2.5	0.773
Peak VO2 (mL/min)	1582 ± 386	1745 ± 609	0.424

Abbreviations: AF, atrial fibrillation; BMI, body mass index; CHADS2VASc score, congestive heart failure, hypertension, age, diabetes, stroke risk score; HR, heart rate; LAVI, left atrial volume index; LVEDVi, indexed left ventricular end diastolic volume; LVEF, left ventricular ejection fraction; LVESVi, indexed left ventricular end systolic volume; MLWHQ, Minnesota Living With Heart Failure Questionnaire score; NT‐proBNP, N‐terminal pro B‐type natriuretic peptide; VO2, oxygen uptake.

### 3.3 Catheter Ablation

All participants underwent CA, with eight (15.1%) undergoing repeat CA for AF or AT recurrence before follow‐up evaluation. A total of 49 (80.3%) procedures were performed under sedation and 12 (19.7%) under general anaesthetic. Pulmonary vein isolation was achieved in 52 (98.1%) participants, and additional ablation was performed in 15 (28.3%). One patient did not complete follow‐up due to re‐location after CA, and one patient died of HF 5 months after an uncomplicated CA. Four (7.5%) participants had a recurrence of persistent AF/AT after their final procedure and were not in sinus rhythm at follow‐up.

### 3.4 Cardiac Function Parameters After CA

At repeat assessment 6 months after CA, LVEF was greater (34 ± 9% vs. 52 ± 9%, *p* < 0.001), and NT‐proBNP had significantly reduced [202 pg/mL (101, 399), *p* < 0.001]. There was no significant change in LAVi. Participants improved their HF symptom burden by MLWHQ score [11.0 (3.3, 34.3), *p* < 0.001]. Fewer participants were on beta‐blockers at follow‐up [52/53 (98.1%) vs. 44/51 (86.3%), *p* = 0.03] with no significant difference in other HF medications. No interval cardiac devices were implanted.

### 3.5 EOV Responders

Eight (80%) participants who demonstrated an EOV pattern at baseline did not after CA. They had a reduction in EOV burden (71.1 ± 11.9% to 48.8 ± 14.8%, *p* = 0.006) during the test and in cycle amplitude [2.6 L/min (2.5, 3.2) vs. 2.2 L/min (1.8, 2.6), *p* = 0.028]. The change in CL was not significant (*p* = 0.05).

The EOV pattern persisted in two (20%) participants despite restoration of sinus rhythm. Both participants had a known alternative cause of HF. One had pre‐existing non‐ischaemic cardiomyopathy and normalized LVEF in sinus rhythm, whereas the other had ischaemic cardiomyopathy and remained in severe LVSD in sinus rhythm.

### 3.6 Other Metabolic Parameters After CA

Participants achieved a greater peak VO2 (1714 ± 574 mL/min vs. 1888 ± 591 mL/min, *p* = 0.003) after CA, and the incidence of ventilatory inefficiency was lower [25 (48.1%) vs. 16 (34.0%), *p* = 0.004]. The maximum heart rate achieved during the test was lower in sinus rhythm (139.4 ± 18.5 bpm vs. 119.9 ± 21.6 bpm, *p* < 0.001), and the oxygen pulse increased (12.3 ± 3.8 mL/beat vs. 15.7 ± 4.0 mL/beat, *p* < 0.001).

**Figure 2:**
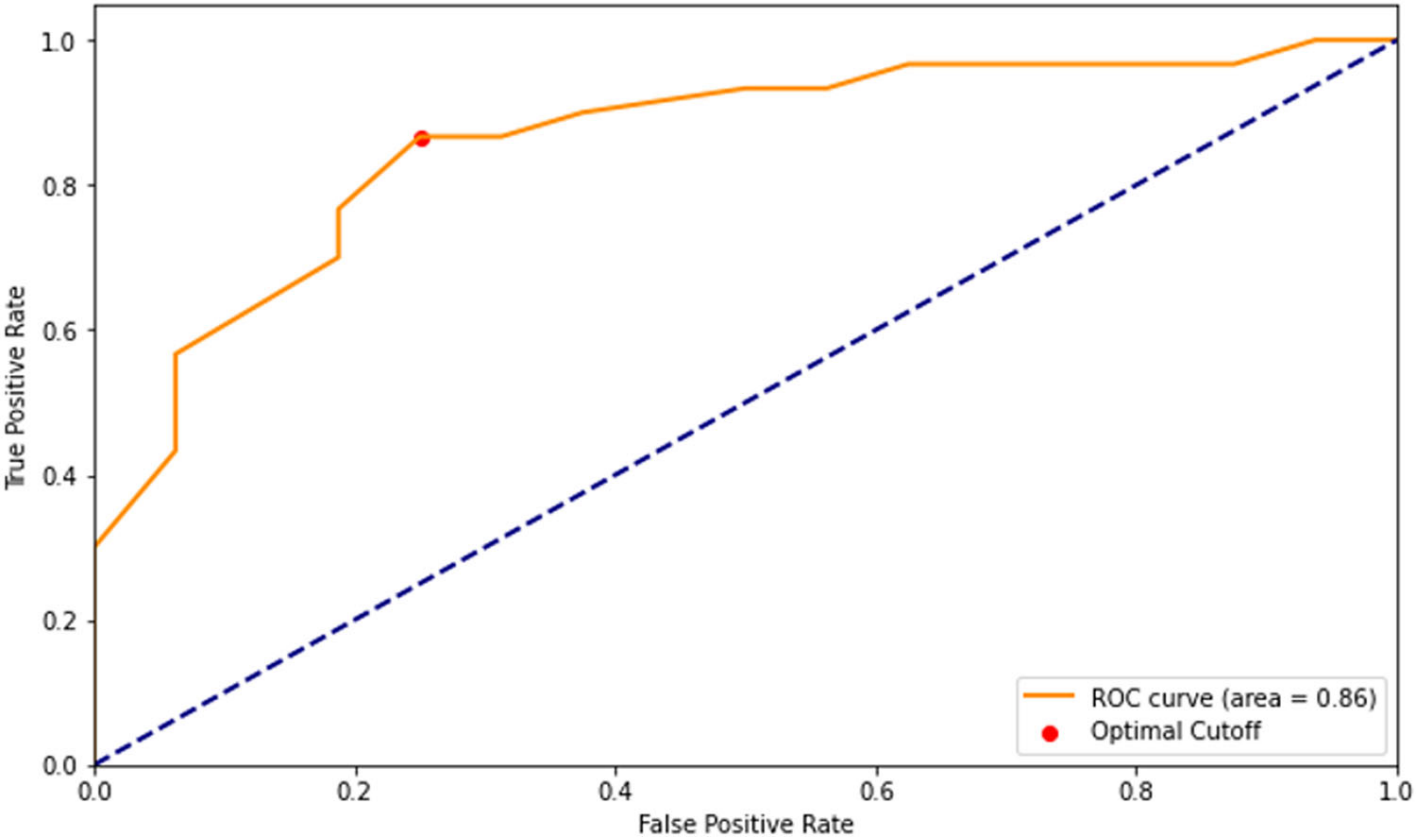
Receiver operating curve of the PetCO2 at peak exercise on baseline test for predicting normalization of VE/VCO2 slope gradient after catheter ablation.

P_ET_CO_2_ at peak exercise also increased after CA (33.7 ± 6.1 mmHg to 41.2 ± 5.8 mmHg, *p* < 0.001). The change in peak P_ET_CO_2_ correlated with the change in HF symptom burden by MLWHQ (*r* = −0.44, *p* = −0.003) and correlated with the change in objective markers of HF; LVEF (*r* = 0.43, *p* = 0.003) and peak VO2 (0.42, *p* = 0.003). The baseline P_ET_CO_2_ could predict the change in MLWHQ score after CA on linear regression analysis (*R* ^2^ = 0.09, *p* = 0.04). It had an AUROC of 0.86 for predicting normal VE/VCO2 at follow‐up (Figure 2).

## 4 Discussion

**Figure 3:**
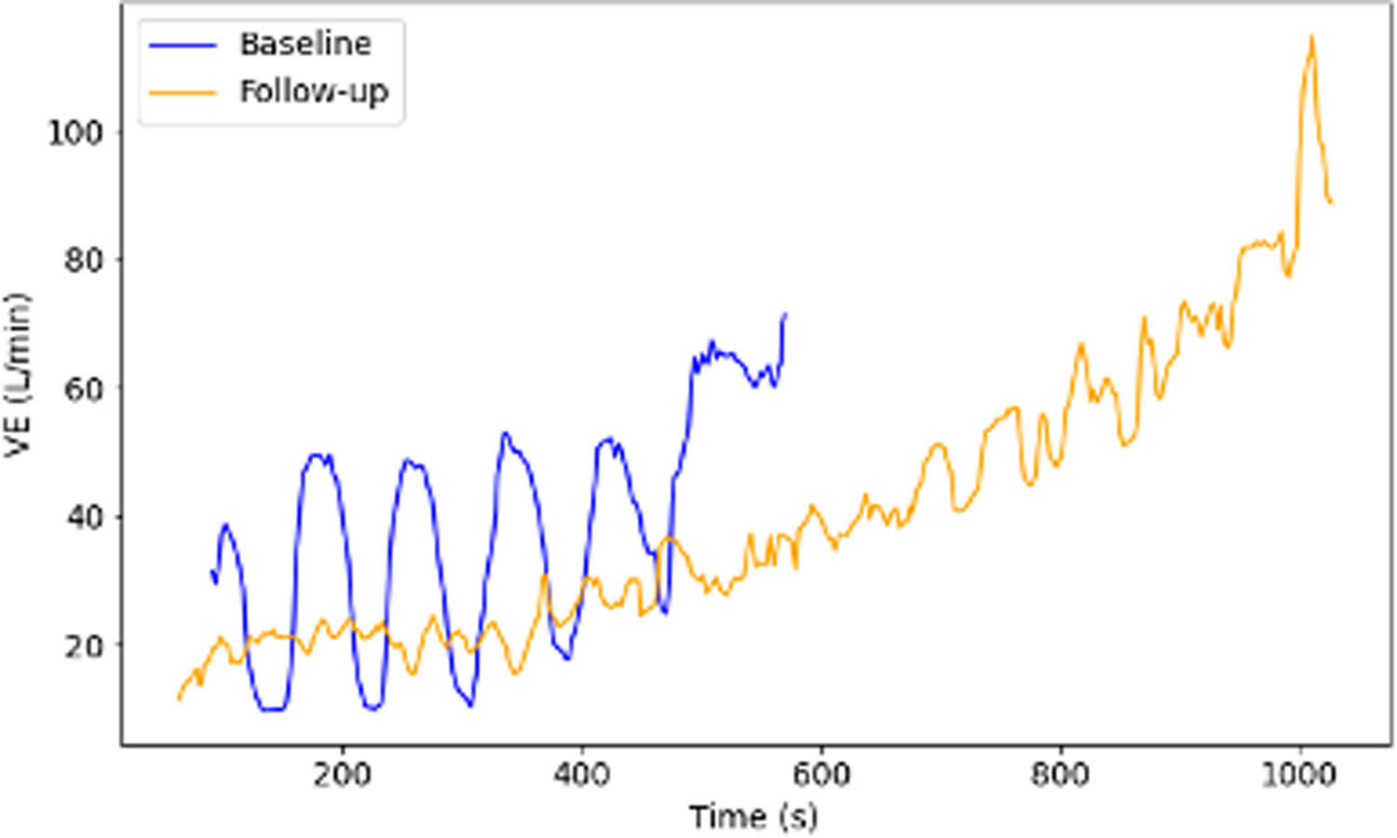
Plots of raw minute ventilation (Ve) values throughout the exercise phase of baseline (blue) and follow‐up (orange) exercise tests from the same participant.

Ventilatory inefficiency is a hallmark of advanced HF and is associated with a poor prognosis [7]. In our cohort of 53 patients with persistent AF with reduced LVEF undergoing CA, 10 (19.2%) exhibited EOV. This prevalence is at the upper end of the reported range of other patient populations with LVSD (7%–25%) [7, 8]. In the most extensive systematic CPET evaluation of patients with LVSD, 79/1280 (6%) of the cohort had concurrent AF, but the prevalence of EOV in this sub‐group was not reported [8]. A retrospective observational analysis of unselected patients undergoing CPET also reported a higher incidence of AF in patients with EOV than in LVEF‐matched controls (16/47 vs. 6/47, *p* = 0.01) [9]. Our cohort's VE/VCO2 slope gradient was similar to previous reports in unselected HF populations undergoing CPET, although the distribution in patients with concurrent AF has not explicitly been reported previously (Figure 3).

The underlying drivers of EOV are incompletely understood, but an increased ventilatory drive may be influenced by elevated LA pressures and stretch due to pulmonary congestion and volume overload. In our cohort, participants with EOV had larger LA and LV volumes, which may reflect higher LA pressures and chronic activation of stretch receptors. Although reverse remodeling may continue after 6 months, LAVi did not significantly change after CA, and other features contributed to the predisposition to develop EOV in AF.

EOV was first described in a cohort of 31 patients with dilated or ischaemic cardiomyopathy [10]. The severely reduced LVEF was believed to result in delayed transmission to the chemoreceptors, contributing to a lag leading to overcompensation and oscillatory ventilation. However, there was no significant difference in LVEF; previous studies have also reported no association with cardiac index or stroke volume. However, participants also had lower mitral E‐wave velocity, which can reflect LV filling pressures and high pulmonary pressures. This may be an indirect marker of pulmonary congestion associated with ventricular dilation in cardiomyopathies and may contribute to EOV in these patients.

Exaggerated chemosensitivity is also seen in EOV and can lead to the overcorrection of the deviations from the arterial CO2 set‐point, manifesting as an oscillatory ventilation pattern. The P_ET_CO_2_ at peak exercise correlated with the HF symptom burden, commonly as shortness of breath and exercise intolerance. The perceived symptoms may result from this maladaptive response, limiting activity and quality of life. The P_ET_CO_2_ significantly increased after CA and was associated with reduced HF symptom burden. CA may reset the P_ET_CO_2_ set point with greater tolerance for hypercapnia without overcompensation. This may improve homeostatic control, produce smaller amplitude ventilatory deviations, lower the EOV burden, and thus resolve the EOV pattern. Intrinsic factors unaffected by CA may influence the VE CL.

This is the first description of the impact of CA on EOV and the VE/VCO2 slope. EOV resolved after CA in all participants with AF‐induced cardiomyopathy (AIC); patients that normalized their LVEF with no alternative causes for HF. EOV is a reversible phenomenon and responds to the treatment of the underlying driver of HF (Figure 3). Improvement has been previously described after cardiac resynchronization therapy in indicated patients [3]. The VE/VCO2 slope gradient also normalized in nine (19.1%) patients after CA, although the phenomenon persisted in some patients. The improvement is in keeping with the demonstrated prognostic benefit in clinical outcomes after CA in patients with advanced HF with LVSD [11].

EOV is a categorical outcome derived from thresholds applied to continuous parameters. These are based on predictive outcomes retrospectively demonstrated in observational studies. The underlying parameters can be measured in patients who do not meet the criteria for EOV, and they improve after CA, suggesting a potential utility in longitudinal follow‐up and potentially as an objective surrogate for HF symptom burden.

### 4.1 Limitations

The limitations of this work should be noted. Follow‐up CPET was performed at 6 months, and further structural remodeling and change in EOV parameters may occur beyond this time. However, studies have shown an attenuation in the resolution of EOV resulting from pharmacological therapy beyond 6 months [5]. Our cohort size was relatively small, limiting sub‐group analyses. but to our knowledge, this is the largest evaluation of EOV in patients with AF. The relative contribution of AF is difficult to isolate, and a comparative evaluation of patients with AF with preserved LVEF and patients with LVSD without AF would be helpful as controls. A matched control arm of patients that did not undergo CA may also help characterize the specific effect of the intervention.

## 5 Conclusion

Ventilatory Inefficiency is common in patients with AF and LVSD, and EOV is associated with LA and LV size. It improves after successful CA in patients with AF‐induced cardiomyopathy. P_ET_CO_2_ at peak exercise increases after CA and may reflect improved chemosensitivity and homeostasis.

## Conflicts of Interest

R.H. has received research grants and educational grants from Medtronic and Biosense Webster, and speaker fees and travel grants from Abbott and Biosense Webster and Medtronic. R.J.S. has received research grants and educational grants from Abbott and Biosense Webster and Medtronic, and speaker fees and travel grants from Abbott and Biosense Webster and Medtronic. R.H., S.H. and R.J.S. were inventors of the STAR mapping system and are shareholders in Rhythm AI Ltd. The remaining authors have nothing to disclose.

## Data Availability

The data that support the findings of this study are available from the corresponding author upon reasonable request.
